# Thymol and Thyme Essential Oil—New Insights into Selected Therapeutic Applications

**DOI:** 10.3390/molecules25184125

**Published:** 2020-09-09

**Authors:** Adam Kowalczyk, Martyna Przychodna, Sylwia Sopata, Agnieszka Bodalska, Izabela Fecka

**Affiliations:** 1 Department of Pharmacognosy and Herbal Medicines, Faculty of Pharmacy, Wroclaw Medical University, Borowska 211, 50-556 Wroclaw, Poland; adam.kowalczyk@umed.wroc.pl (A.K.); izabela.fecka@umed.wroc.pl (I.F.); 2 Student’s Scientific Group of Department of Pharmacognosy and Herbal Medicines, Faculty of Pharmacy, Wroclaw Medical University, Borowska 211, 50-556 Wroclaw, Poland; martyna.przychodna@student.umed.wroc.pl (M.P.); sylwia.sopata@student.umed.wroc.pl (S.S.)

**Keywords:** thymol, thyme essential oil, new therapeutic applications

## Abstract

Thymol (2-isopropyl-5-methylphenol) belongs to the phenolic monoterpenes and mostly occurs in thyme species. It is one of the main compounds of thyme essential oil. Both thymol and thyme essential oil have long been used in traditional medicine as expectorant, anti-inflammatory, antiviral, antibacterial, and antiseptic agents, mainly in the treatment of the upper respiratory system. The current search for new directions of biological or therapeutic activities of natural plant substances with known structures includes thyme essential oil and thymol. Novel studies have demonstrated their antibiofilm, antifungal, antileishmanial, antiviral, and anticancer properties. Also, their new therapeutic formulations, such as nanocapsules containing these constituents, can be beneficial in medicinal practice and create opportunities for their extensive use. Extensive application of thymol and thyme essential oil in the healthcare sector is very promising but requires further research and analysis.

## 1. Introduction

The genus Thymus from the Lamiaceae family contains many representatives. These plants, originating from the Mediterranean area, are commonly used for food, cosmetic, and medicinal purposes [1]. A thyme herb obtained from *Thymus vulgaris* L. and *Thymus zygis* L. is the most well-known herbal substance in the pharmaceutical industry. Nowadays, only standardized preparations of thyme herb and essential oil that meet the requirements of national pharmacopeias or European Pharmacopoeia X (Ph. Eur. X) are used for the production of medicines.

According to the Ph. Eur. X definition, thyme herb is described as whole leaves and flowers separated from the dried stems of *T. vulgaris* or *T. zygis* or their mixture with 12 mL/kg of minimum essential oil (EO) and minimum thymol and carvacrol contents of 40% [2]. Thyme EO is defined as a product of the steam distillation of fresh flowering aerial parts of one or a mixture of both species with 37–55% thymol and 0.5–5.5% carvacrol concentrations [2,3].

The thyme herb, EO, and their main volatile components—thymol and carvacrol—have found wide application for therapeutic objectives. The scope of medical usage, preclinical, and clinical data, as well as the chemical composition of thyme herb and EO, have been summarized and published by the Committee on Herbal Medicinal Products (HMPC) of the European Medicines Agency (EMA) as the corresponding assessment reports and monographs [3,4]. When discussing the chemical composition and activity of thyme herb and its hydroalcoholic extracts, except for the volatile fraction, non-volatile components such as flavonoid glycosides, caffeic acid oligomers, simple phenolic acids, hydroquinone derivatives, and terpenoids should also be taken into account. Detailed information about these components can be found in the literature [5,6,7].

**Figure 1:**
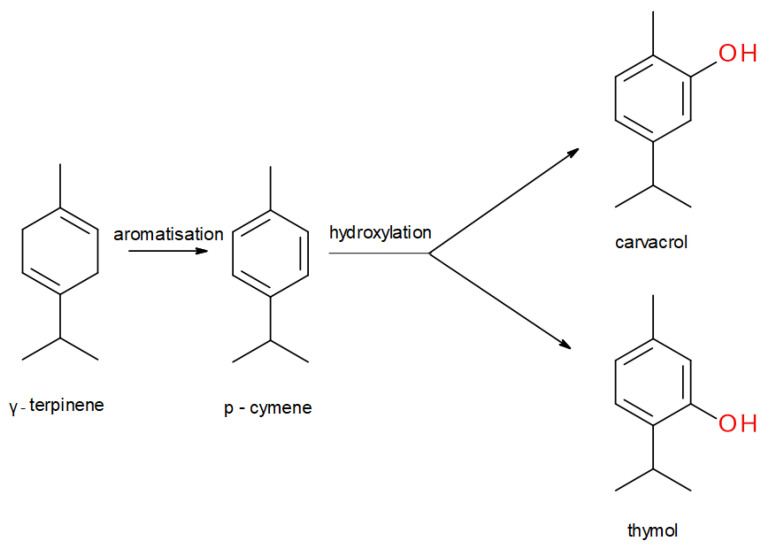
Scheme of the key steps of thymol and carvacrol biosynthesis.

**Figure 2:**
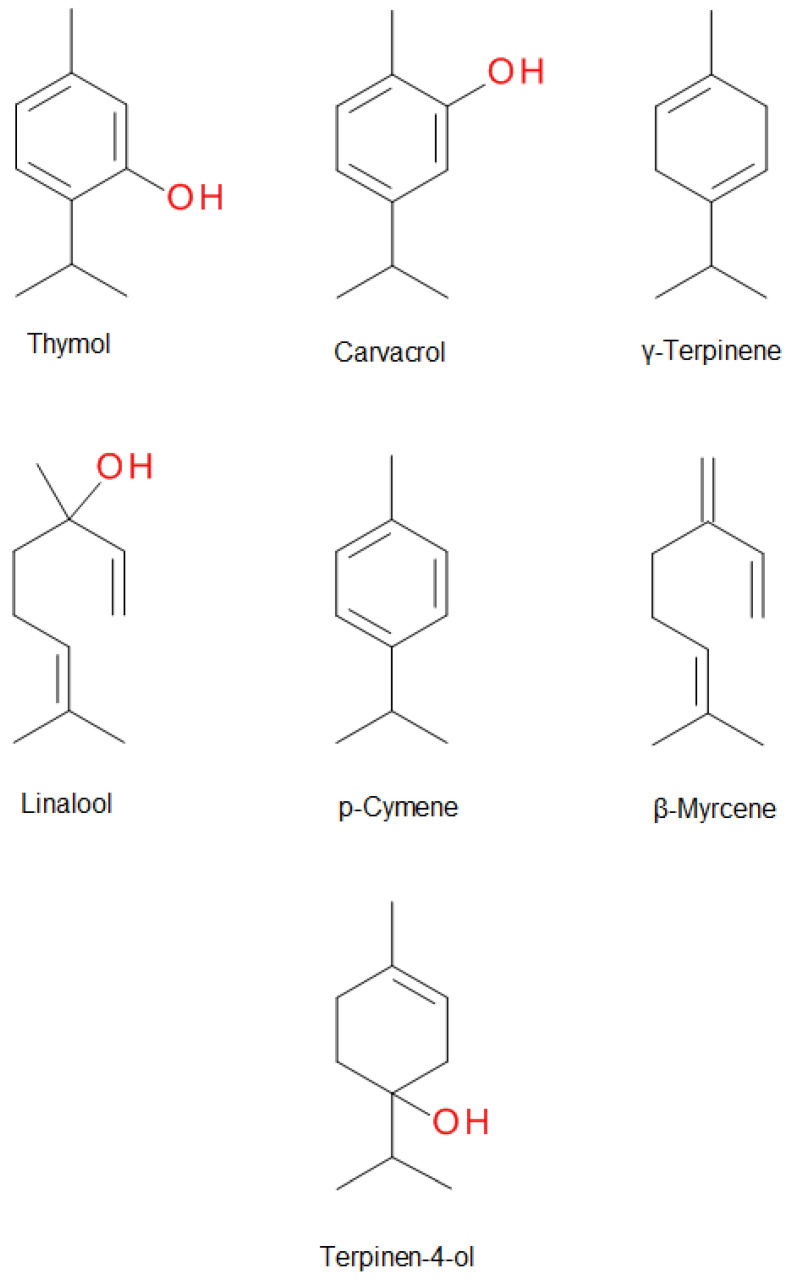
The structure of the main components of thyme essential oil.

**Figure 3:**
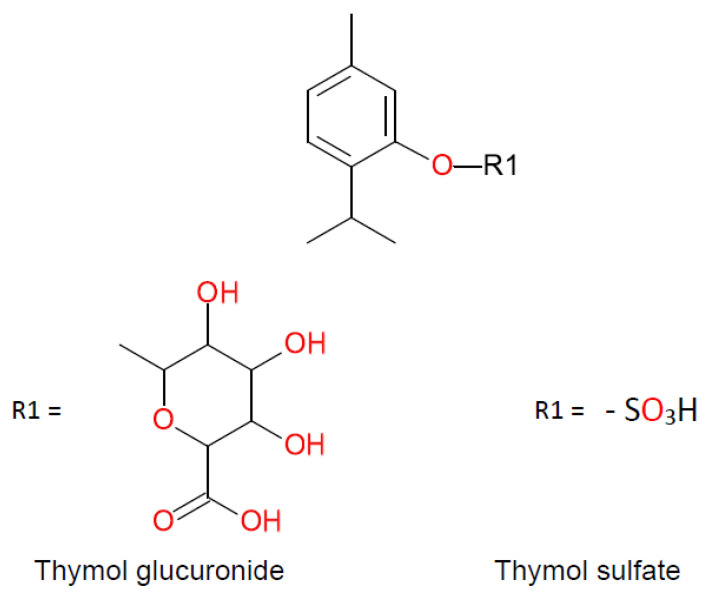
Thymol metabolites.

Thyme EO compounds belong to various chemical groups including monoterpenes, monoterpene alcohols, phenol derivatives, ketones, aldehydes, ethers, and esters. There are also numerous chemotypes within the *T. vulgaris* species, differing in the main component of EO, but only “thymol type” with thymol as the main constituent is listed in the European Pharmacopoeia. The main components of thyme EO are the isomeric phenolic monoterpenes thymol (2-isopropyl-5-methylphenol) and carvacrol (2-methyl-5-(propan-2-yl)phenol). Both these monoterpenes are biosynthesized by the hydroxylation of p-cymene after the aromatization of γ-terpinene to *p*-cymene. The scheme of the biosynthesis pathway is shown in Figure 1 [6]. Thymol is a colorless, crystalline compound with characteristics including strong odor and solubility in alcohol and other organic solvents, but it is only slightly soluble in water [8]. Carvacrol, on the other hand, is a colorless to pale yellow liquid, insoluble in water but highly soluble in ethanol, acetone, and diethyl ether and with a thymol odor [9]. Structures of thymol, carvacrol, and other thyme EO components—*p*-cymene, γ-terpinene, linalool, β-myrcene, terpinen-4-ol—are shown in Figure 2. Thymol after oral administration is rapidly absorbed and slowly eliminated approximately within 24 h. It is found in the form of thymol sulfate in plasma, and two phase II conjugates—thymol sulfate and thymol glucuronide—can be found in the urine. The formation of glucuronide was observed only at higher doses. Thymol metabolites are presented in Figure 3. Oral bioavailability referred to as thymol sulfate is approximately 16% and the plasma half-life is approximately 1.5 h [10,11]. The bioactivity and toxicological actions of carvacrol were described by Sharifi and co-workers [9].

Thyme herb and its volatile oil have long been used for the treatment of upper respiratory tract infections, symptoms of bronchitis, parasitic infections, pruritus associated with dermatitis, bruises, and sprains. Nowadays, it is generally used as an expectorant in cough associated with cold and also in dentistry as a disinfectant [12]. It exerts an antibacterial effect on Gram-positive and Gram-negative bacteria and has antiviral (herpes simplex virus type I, human rhinoviruses and influenza viruses), antifungal, antioxidant, anti-inflammatory, and spasmolytic activity. Although thyme volatile oil has cytotoxic properties in high concentrations and may cause intestinal cell damage when administered orally, no toxicity has been reported at commonly used doses, and it can be considered as a safe drug. Skin administration in high concentrations may cause irritation. In rare cases, an allergic reaction can occur, manifesting as skin rash, bronchospasm, asthma attack, and anaphylaxis. Therefore, this EO is contraindicated in persons allergic to thyme or other plants from the Lamiaceae family due to a possible cross-reactivity [3,13,14].

Thymol, as the main active ingredient responsible for the activity of thyme EO, has been shown to possess antiseptic, antibacterial, antifungal, anthelmintic, antiviral, antioxidant, expectorant, antispasmodic, carminative, diaphoretic, sedative, anti-rheumatic, and even anti-cancer, anti-hyperlipidemic and anti-hyperglycemic action [6,15,16,17,18,19]. The research into substances with new biological and pharmacological activities includes commonly known natural substances, including thymol and thyme EO.

This review presents selected directions of action and potential possibilities of their use for therapeutic purposes—more recent reports, in addition to the HMPC publications. In particular, our review contains data on infectious and neoplastic diseases, which are a challenge for modern medicine. Our area of interest is also related to newer pharmaceutical forms that increase the bioavailability and activity of thymol and thyme oil.

## 2. Activity against Microbiological Biofilms

Antibiotic resistance to currently used and frequently overused antibiotics is a growing problem in the pharmacotherapy of bacterial infections. Therefore, it is very important to search for new substances, including those of natural origin, that can be used to combat pathogens [20,21]. Bacterial and fungal resistance to antibiotics and disinfectants is related to their ability of biofilm production that prevents the penetration of the antibacterial agent into the site of infection. Biofilm is defined as a group of microorganisms connected by a cell-free polymer matrix composed mainly of polysaccharides, protein, and DNA. This structure shows adhesion to solid surfaces, usually internal implants, valves, or catheters. Pathogenic bacteria and fungi are protected by a thick layer of polysaccharide against harmful factors, such as immune cells, oxidative stress, and antimicrobial agents. These microorganisms are a thousand times more resistant to antimicrobials than planktonic forms. This is caused by the physiological differences of cells that make up the biofilm—their metabolic rate and oxygen and nutrient consumption are lowered. These mechanisms lead to increased tolerance to commonly used antibiotics and exacerbation of infectious processes [22,23,24]. In addition, the presence of biofilm facilitates the communication of bacteria and fungi by quorum sensing (QS). This is a communication system between bacterial cells affecting the regulation of gene expression in response to the density of microbial populations. QS is of particular importance in the colonization of new territories by bacteria. It specifies the conditions to which individual microorganisms can adapt [25,26]. It is estimated that 65–80% of all infections are caused by biofilm-related microorganisms, which is the main reason for the ineffectiveness of treatment [27].

According to the WHO, priority bacteria that are also able to form biofilm include carbapenem-resistant *Pseudomonas aeruginosa*, *Staphylococcus aureus*—especially methicillin-resistant (MRSA)—and third-generation cephalosporin-resistant *Enterobacteriaceae* [28]. Infections caused by the above-mentioned microorganisms, due to their ability to produce virulence factors, are a serious problem of modern pharmacotherapy. Based on the Antibiotic Resistance Report of 2019, over 2.8 million infections with antibiotic-resistant bacteria occur in the US every year, causing deaths of over 35,000 people [29]. Another challenge for modern antibiotic therapy is the high toxicity of currently used antimicrobial medications and, consequently, the severe side effects they cause. Often therapeutic doses are too cytotoxic to be used in the treatment [30]. Research indicates that natural bactericides can be an effective alternative to commonly used synthetic or semisynthetic antimicrobial drugs.

Thyme EO antimicrobial activity depends on the percentage composition of its main components [31]. EOs with a high phenolic monoterpene percentage, primarily thymol, have the strongest antibacterial properties connected with their structure. Small and lipophobic particles can easily overcome lipid barriers [32]. Pathogens’ cell membrane, due to its hydrophobic composition, is a target for EO compounds. Studies on the mechanism of thymol’s antibacterial activity indicate that its ability to integrate into the lipid layer of the cell membrane increases the surface curvature. The hydrophilic part of the molecule interacts with the polar part of the membrane, while the hydrophobic benzene ring and aliphatic side chains sink into the inner part of the biological membrane. This causes large changes in membrane structure by destabilization of the lipid layer, elasticity decrease, and fluidity increase. This process leads to increased permeability to potassium and hydrogen ions. It also affects the activity of internal membrane proteins such as enzymes and receptors. After incorporation into the cell membrane, thymol interacts with its embedded proteins through various non-specific mechanisms, which lead to changes in the conformation and activity of internal and membrane proteins. Thus, cell membrane tension and destabilization can be induced by the presence of thymol. Carvacrol acts on the bacterial membrane in a similar way to thymol. Isolated compounds have stronger destructive effects than EO which contain them [33,34,35,36].

**Figure 4:**
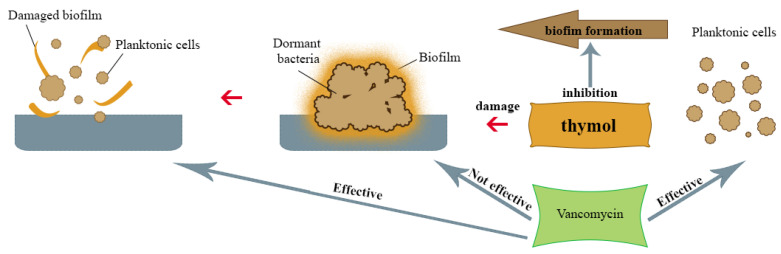
Possible mechanism of antibiofilm action of thymol.

Kryvtsova and co-workers [31] studied the effect of *T. vulgaris* EO on various typical bacterial strains, including (MRSA) methicillin-resistant *S. aureus* isolated from the oral cavity of patients suffering from periodontitis and pharyngitis. The test of antibiofilm activity showed that the bacteria biofilm was reduced by 53% after exposure to the lowest concentration of thyme EO (0,01% *v*/*v*). Higher concentration usage (0.05% *v*/*v*) led to a structure reduction of 76%. The lowest activity was demonstrated against *Streptococcus pyogenes* and *Escherichia coli*. The main components of volatile oil used in this research were phenolic monoterpenes, including thymol. Also, Tohidpour and co-workers [37] investigated the effect of *T. vulgaris* and *Eucalyptus globulus* Labill. EO on clinically isolated MRSA. It was found that thyme EO, which contained thymol as a main ingredient, showed better growth-inhibiting properties for these microorganisms than eucalyptus EO. Zhongwei and co-workers [38] also evaluated the effect of thymol on MRSA biofilm and the potential synergy of thymol with vancomycin, which is the antibiotic of choice for this pathogen infection. Thymol has shown the ability to inhibit biofilm formation and eliminate the mature MRSA biofilm by inhibiting the synthesis of important biofilm components of these bacteria, such as polysaccharide intercellular adhesin (PIA) and extracellular DNA (eDNA), which is an important mechanism for *S. aureus* biofilm formation. These structures are responsible for, among other functions, supporting cell aggregation and adhesion to neutral solid surfaces, which determines antibiotic resistance [39,40]. Neither thymol nor vancomycin applied to MRSA colonies in the biofilm at the concentrations used showed complete bacterial elimination; however, in the combination of these compounds, the percentage of live bacteria was significantly reduced (Figure 4), which suggests that thymol is effective in supporting the therapeutic effect of vancomycin on MRSA biofilms. In 2019, Kerekes and co-workers [41] studied potential antimicrobial inhibition of biofilm formation and the quorum-sensing mechanism (QS) by Ceylon cinnamon tree (*Cinnamomum zeylanicum* Blume), marjoram (*Origanum majorana* L.) and thyme (*T. vulgaris*) EO and three main selected compounds from them—trans-cinnamaldehyde, terpinen-4-ol and thymol. Among them, thymol showed the most potent activity against single-species biofilms of *E. coli*, *Listeria monocytogenes*, *Pseudomonas putida* and *S. aureus*. In a study on polymicrobic bacterial cultures, *T. vulgaris* EO showed a strong biofilm-reducing effect on *E. coli* and *L. monocytogenes* at a concentration of 0.5–4 mg/mL. On a dual-species (*L. monocytogenes* with *S. aureus*) biofilm thyme EO caused growth reduction at a concentration of 0.2–1.5 mg/mL and bacterial death at a concentration of 0.2 mg/mL. The destructive effect of thyme EO and thymol on *E. coli*, *S. aureus*, *L. monocytogenes* and *P. putita* was also observed in a study using the scanning electron microscope (SEM) [42]. Research conducted by Qaralleh [30] showed a strong biofilm-reducing effect of *T. capitata* L. EO on *P. aeruginosa*, which was probably also associated with the presence of thymol as a main EO component. Biofilm reduction of 44.8% and 49.8% was achieved at concentration of 0.0046% (*v*/*v*), while 99.6% and 98.4% reduction (depending on the *P. aeruginosa* strain) were observed at a concentration of 0.041% (*v*/*v*).

*Klebsiella pneumoniae*, from the *Enterobacteriaceae* family, is the cause of numerous chronic hospital infections with high mortality and prolonged hospitalization due to the high virulence of this pathogen, dynamically developing antibiotic resistance, and limited treatment methods [43]. In a study conducted by Mohamed [44] on biofilm-forming *K. pneumoniae* strains, the biofilm eradication ability from 80.1 to 98.0% thyme EO in different concentrations was demonstrated. The main component responsible for these antibacterial properties was thymol. Pure thymol (5 µg/mL) has also been tested and its biofilm reduction ability was confirmed. The capacity to reduce pathogen viability, however, was not demonstrated. Strong synergism in action on cell viability has been observed using thyme EO and thymol with ciprofloxacin. The use of the EO allowed the dose of the antibiotic to be lowered in both biofilm-forming and plankton cells. The study results suggest that the combination of thyme EO or thymol with this antibiotic could reduce its possible toxic effects and the cost of treatment.

## 3. Antifungal Activity

### 3.1. Thymol Activity in Cryptococcosis

Cryptococcosis is a systemic mycosis caused by fungi of the genus *Cryptococcus*, most often by the species *C. neoformans* [45]. In the last decade, the percentage of *C. gattii* infections has increased, while infections with *C. albidus* and *C. laurentii* species are rare. [46]. This pathogen mainly attacks the respiratory and nervous systems, causing pneumonia or meningitis [45,47]. This disease mainly affects immunocompromised patients, especially those with AIDS [48]. Over 223,000 cryptococcal meningitis cases occur worldwide each year, of which approximately 181,000 are fatal [46]. This infection is often associated with patient catheterization, artificial heart valves, or dialysis [49,50]. Treatment of such an invasive disease is problematic because of the ability of *C. neoformans* and *C. laurentii* to produce a polysaccharide-rich extracellular biofilm matrix. This makes cryptococcal cells resistant to standard treatments such as azole antifungal agents. Amphotericin B is effective in the treatment of *Cryptococcus* sp. infections, but concentrations against fungal biofilms exceed maximum doses, which may lead to toxic symptoms, mainly renal impairment. Biofilm-associated fungal cells are protected against macrophage phagocytosis, which increases their resistance. Therefore, it is important to look for new active substances against *Cryptococcus* strains. Kumari and co-workers [51] investigated the antifungal activity of various components of Lamiaceae EO against *C. neoformans* and *C. laurentii* planktonic cells. Six active ingredients were used: thymol, carvacrol, eugenol, citral, cinnamaldehyde, and menthol. Thymol and carvacrol proved to have the best antifungal effect, which inhibited both planktonic *Cryptococcus* species’ cells, biofilm formation, and growth. The results of this study were compared with standard therapy with amphotericin B, nystatin, and fluconazole. These drugs showed antifungal activity against planktonic *Cryptococcus* forms in lower minimum inhibitory concentration (MIC) in comparison to thymol and carvacrol. However, the biofilm-eradicating concentrations of those drugs were 32–64 times higher than the MIC for planktonic forms, while thymol and carvacrol were effective for biofilm reduction at concentrations 4–8 higher than MIC. Therefore, it was concluded that *C. neoformans* and *C. laurentii* biofilm was much more sensitive to thymol or carvacrol compared to standard therapies. The study also revealed that the biofilm of *C. laurentii* was more susceptible to the active ingredients of the EO compared to *C. neoformans*. This has been confirmed by scanning electron microscopy (SEM) images of thymol-treated fungal colonies. A significant reduction in the number of cells together with a deformed and perforated outer cell membrane caused by penetration of thymol into the outer layer of the cell membrane leads to the expansion of dipalmitoylphosphatidylcholine (DPPC). This reduces membrane elasticity and disrupts the lipid layer, which causes cell death via a rapid outflow of intracellular components [52].

### 3.2. Other Antifungal Properties

**Figure 5:**
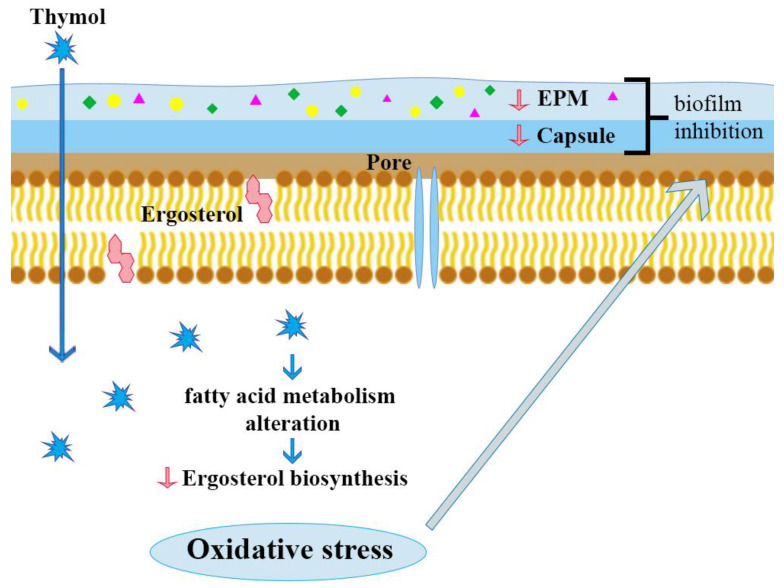
Possible mechanism of antifungal action of thymol.

Ergosterol is a unique sterol found only in the cell membrane of fungi, important for their proper growth and functioning. Therefore, compounds affecting its level may have antifungal activity. The probable antifungal mechanism of thymol is based on the effect on fatty acid metabolism, including ergosterol in the fungal cell. It leads, among other effects, to an increased concentration of reactive oxygen species and oxidative stress, which causes a decrease of the extracellular polymer matrix (EPS) and capsular polysaccharide (Figure 5). Ergosterol decrease was observed in cell membranes of *Candida* and also *Cryptococcus* treated with thymol, which caused disruption of membrane integrity, membrane-associated enzyme disturbances, extensive damage and, as a consequence, cell death [34,53,54]. Al-Shahrani and co-workers [55] studied the antifungal activity of *T. vulgaris* EO on clinical strains of *Fusarium* spp., *Aspergillus* spp. (*A. flavus*, *A. niger*), and *Candida* spp. (*Candida albicans*, *C. glabrata*, *C. kefyr* and *C. parapsilosis*). As a reference, amphotericin B was used. The results confirmed that this EO has strong antifungal properties against all strains in concentrations ranging from 0.5 to 10 mg/mL. Therefore, it can be an effective alternative to currently used antifungal medications such as amphotericin B, which have high toxic potential.

Caprylic acid is one of many saturated fatty acids found in lipids of plant and animal origin. Thymol or carvacrol in combination with caprylic acid has synergistic antifungal activity [56]. The mechanism of antifungal activity is associated with the ability of caprylic acid to be incorporated into the lipid layer, which increases the fluidity of the fungal cell membrane, disrupts its structure and function, and causes conformational changes in membrane proteins, the release of intracellular components, and cell disintegration [57]. In a study conducted by Bae and Rhee [58] the elimination of *C. albicans* was obtained during a few minutes’ of exposure to caprylic acid (1–1.5 mM) with thymol or carvacrol (0.5–1.5 mM). This combination made it possible to reduce the dose of caprylic acid, and thus possible toxic effects. The synergism of these combinations may cause the increased passage of these phenolic monoterpenes into the cell as caprylic-acid-induced fungal membrane disruption. Thus, this kind of combination could be used in the treatment of fungal infections caused by *C. albicans*.

## 4. Antileishmanial Properties

Parasitic infections are still a serious health problem, especially in less developed countries, where poor hygiene, overpopulation, and migration of people from neighboring countries are common problems. The development of parasites is also favored by a humid and hot tropical climate [59]. Leishmaniasis is caused by flagellate protozoa of the genus *Leishmania*. According to the WHO, there are between seven hundred thousand and one million new cases of this disease annually [60]. Therefore, it is crucial to look for new and readily available antiparasitic agents. The parasite *Leishmania infantum* causes visceral leishmaniasis, a vector born disease that mainly affects people from underdeveloped countries. Currently used medications are highly toxic and require long-term treatment. A study conducted by Youssefi [61] revealed the activity of thyme EO ingredients against this parasite in comparison to standard medicine Glucantime (Meglumine antimoniate). An inhibitory effect on *L. infantum* growth after thymol and carvacrol use was demonstrated. In vivo studies have shown in groups treated with thymol not only a decrease of the presence of *L. infantum* in the liver, but also noticeably less hepatocyte necrosis compared to other groups studied. Cytotoxicity studies have shown that thymol was a safer substance than other tested monoterpenoids. The mechanism of thymol activity, as in the case of bacteria or fungi, is probably based on its lipophilic nature, as it can easily be incorporated into cell membranes and disrupt the phospholipid bilayer. Increased permeability of the external and internal membranes leads to an outflow of cellular components and apoptosis. It also affects numerous enzymes responsible for regulatory processes in microbial cells, such as dihydrofolate reductase, necessary for the synthesis of active folic acid, and ATPase, responsible for cell membrane permeability regulation. However, the actual mechanism of action of thymol and other EO components for these parasites has not been confirmed yet. Thymol and carvacrol appear to be promising, safe, and effective alternatives to existing medicines used against *L. infantum*.

## 5. Antiviral Activity

**Figure 6:**
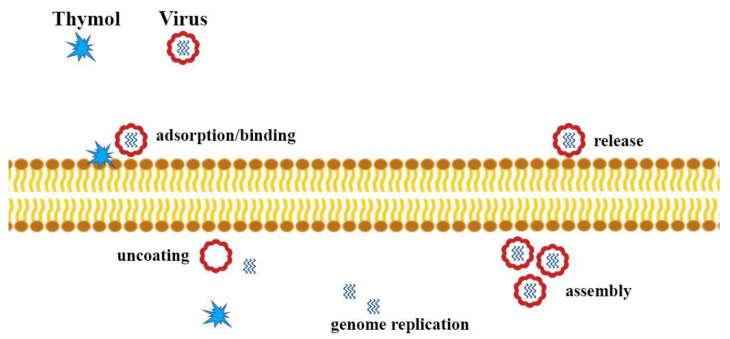
Possible mechanism of antiviral activity of thymol.

Viral diseases continue to be a significant health problem worldwide. It is noted that the relatively narrow mechanisms of action of antiviral drugs lead to the development of viruses’ resistance to commonly used medicines. The mechanisms of action of essential oils (EOs) are versatile due to the complexity of composition and synergism of bioactive compounds compared to synthetic antiviral substances (e.g., acyclovir) that act in a manner specific to a particular type of virus [62]. The action of EOs can interfere with the extracellular level, i.e., the penetration of the virus into the host cell, interfering with the structure of the viral envelope, or block viral proteins that are necessary for the virus to enter the host cells. EOs can also have antiviral effects against intracellular viruses (Figure 6). The mechanisms are not yet fully understood. It is possible to determine at what stage of the infection EOs work, but there are no precise data on the molecular mechanism of action, including specific sites of action, and the types of interactions. It is important to learn about these mechanisms in order to be able to use the potential of EOs in viral infections [63].

### 5.1. Activity Against SARS-Cov-2

One of the biggest challenges facing scientists from all over the world today is to find and develop effective preparations to fight Sars-CoV-2. Intensive efforts are underway to find a vaccine or a cure for the disease it causes. Already used and FDA-approved drugs with different therapeutic indications are being re-examined for their possible use against SARS-CoV-2 [64]. In the meantime, alternative treatments are also being tested. For this purpose, it uses, for example, natural compounds present in EO, which have a wide range of biological effects, including antiviral activity and molecular docking techniques. Molecular docking is a method that allows analysis of the molecular recognition of one molecule by another, and thus the prediction of the ways of binding and affinity to binding in a complex formed by two or more molecules with known structures. Protein-ligand docking is an important type of molecular docking used in modern structure-based drug design. It can be understood as an effective and economical alternative to the experimental methods of determining or predicting the ways of bonding and affinity to bond in a complex between the protein receptor and ligand. Several compounds found in EOs, including thymol, have been studied in this way. For this purpose, the recently determined crystal structure of the SARS-CoV-2 spike glycoprotein (S) was used [65]. The S protein consists of two units. The first is S1, also called a receptor-binding domain (RBD), which binds to the peptidase domain of angiotensin-converting enzyme 2 (ACE2), initiating virus attachment to the host cell surface. The second unit is S2, responsible for the virus-host membrane [66]. The S1 subunit is of great clinical importance as inhibition of RBD may lead to conformational changes of S-protein, which is the first step to hinder viral infections. Studies have shown that carvacrol, anethole, cinnamaldehyde, L-4-terpineol, and others displayed a better binding affinity with high docking scores compared to the rest of the tested ligands. This was associated with better binding affinities of these ligands with the RBD of the S protein by H-bond and hydrophobic interactions with the amino acid residues in the binding site. Thymol, camphene, pulegone, ocimene and menthol also showed good binding affinities. However, they lacked hydrogen binding interactions with the protein. Since there are many hydrophobic residues around the ligands in the binding pocket, hydrophobic interactions between them may contribute to the stability of the complex protein-ligand [65,67]. Both the United States Environmental Protection Agency (EPA) in the USA and the Government of Canada have placed on the list of disinfectants with evidence for use against COVID-19 preparations with thymol as the active ingredient. These products are designed to disinfect external hard surfaces and hands in healthcare, institutional, or residential applications [68,69].

### 5.2. Other Antiviral Properties

Feriotto et al. [70] demonstrated the antiviral activity of three *T. vulgaris*, *Cymbopogon citratus* (DC.) Stapf and *Rosmarinus officinalis* L. EOs against HIV-1. The resulting EOs disturbed the function of the Tat protein, which plays a key role in the HIV replication cycle and can therefore be an important target for antiviral agents. It is a transcription transactivator whose effect is to increase the affinity of the P-TEFb complex kinase for the short TAR viral RNA (trans-activating region) and, consequently, leads to an increase in extended viral transcripts and exit of the latent phase. There was a clear inhibitory potential for EOs obtained from *R. officinalis* (in concentrations from 0.25 to 0.5 μg/mL), *T. vulgaris* (from 3 to 6 μg/mL) and *C. citratus* (from 6 to 12 μg/mL) for binding Tat to TAR RNA. A virus transcription inhibiting effect was also observed. *T. vulgaris* and *C. citratus* EOs reduced HIV-1 transcription by 52% and 60%, respectively, at 0.30 ng/mL and 0.65 ng/mL used. 

Rhinoviruses and influenza viruses are responsible for the majority of acute respiratory infections. Preventive or curative measures for rhinovirus infections do not exist, and despite the availability of flu medications, in fact, treating upper and lower respiratory tract infections often involves using drugs that alleviate symptoms. One of the herbal medicines used to relieve symptoms is thyme extract. Walther and Schmidtke [13] conducted an in vitro study of the effectiveness of fluid thyme extract (R&R Extrakte GmbH; Cologne, Germany), against two strains of rhinovirus and influenza A virus. Thyme extract did not show anti-rhinovirus activity, but showed antiviral activity against the influenza virus. Thyme extract at non-cytotoxic concentrations of 0.03–0.33% *v*/*v* reduced the cytopathic effect by influenza, depending on the dose.

Toujani et al. [71] demonstrated *T. capitatus* activity against HSV-1 and HSV-2 herpes viruses. Ethanol extract (EE) proved to be the most active against both tested viruses. The three most active compounds from *T. capitatus* EE were isolated: β-sitosterol, cinnamaldehyde, and carvacrol. Of these compounds, β-sitosterol showed the strongest antiviral activity. The potential mechanism of action of the extracted active compounds is mainly the direct inactivation of extracellular HSV-2 virions and, as a consequence, the reduced ability of the virus to spread between cells.

## 6. Antineoplastic Activity

Neoplasms (cancer) are a serious global health problem, as they are the second most common cause of death in the world, accounting for approximately 9.6 million deaths in 2018. The incidence of neoplasm is still increasing, so there is a need for new and better treatments [72]. The research directions of common and well-known plant compounds show promising results in creating new antineoplastic substances [73]. Thymol exerts an anti-tumor effect through various mechanisms such as inhibition of cell growth (antiproliferative activity), induction of apoptosis, production of intracellular reactive oxygen species (ROS), depolarization of mitochondrial membrane, activation of Bax proapoptotic mitochondrial proteins, and interaction with caspase or poly-ADP ribose polymerase [74]. Elbe and co-workers [75] studied the antiproliferative and proapoptotic properties of thymol on non-small-cell lung carcinoma, highly metastatic breast adenocarcinoma, and prostate cancer cell lines. Thymol showed cytotoxic effects on the above-mentioned tumor types in a dose-dependent and time-dependent manner. A selective antiproliferative effect against cancer cells was observed. Thymol induced apoptosis of tumor cells, but the mechanism of this action has not been fully explained. It is most likely associated with hydrophobic properties of the thymol molecule and the ability to increase cancer cell plasma membrane permeability.

**Figure 7:**
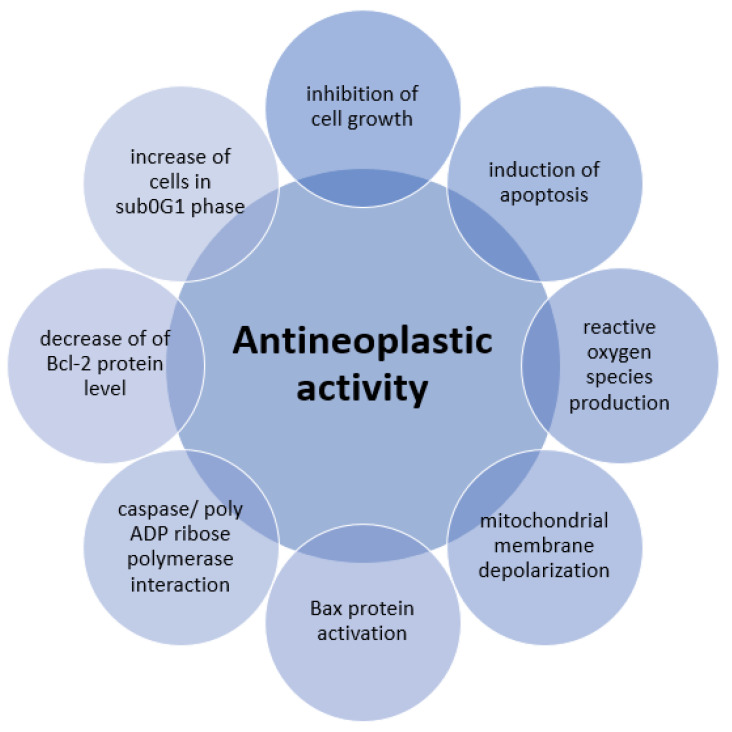
Antineoplastic activity of thymol.

The research team led by Kang [76] found that thymol inhibits cell growth and induces apoptosis in gastric adenocarcinoma cells. The cytotoxic, dose-dependent thymol effect on tumor cells decreased their viability from 89.56 ± 0.84% to 50.75 ± 2.4% after 24 h of exposure to this monoterpene. It was observed that thymol induced significant morphological changes such as lower cell density, fragmentation of cell nuclei, and chromatin condensation. The use of thymol caused an increase in the number of cells in the sub-G1 phase, when the DNA is cleaved, which is a characteristic feature of apoptosis. Another mechanism of thymol cytotoxic activity is the induction of reactive oxygen species (ROS). It caused depolarization of mitochondrial membranes by activating the Bax protein, responsible for the formations of pores in the membrane, and thus the outflow of cytochrome c. Thymol activated caspase degrading protein and led to the activation of poly-ADP-ribose polymerase (PARP), which plays a role in DNA damage detection and its repair, gene stability, and cell death. Also, Günes-Bayir and co-workers [77] confirmed the cytotoxic, genotoxic, and proapoptotic effects of thymol on gastric adenocarcinoma cells. These cell lines were treated with thymol in concentrations of 10, 20, 30, 50, 100, 200, and 400 μM, prepared from a stock solution of 600 μM of thymol in dimethyl sulfoxide (DMSO), and after 24 h of incubation, a dose-dependent decrease in cell survival was observed compared to the control group. Simultaneous measurements of reactive oxygen species (ROS) and glutathione (GSH) levels were performed. ROS induction was also dose-dependent and correlated negatively with cell survival. A reduced amount of GSH was observed in exposed cancer cells at a concentration of 50 μM. Investigation of the effect of thymol at a concentration of 10 to 100 μM on gastric adenocarcinoma cells confirmed its antitumor effect, manifested by apoptosis induction: cell nucleus shrinkage and chromatin condensation. This mechanism is associated with a decrease in the level of Bcl-2 protein and an increase in the level of Bax protein, caspase-3, and caspase-9. A significant proapoptotic effect of thymol was found at a dose of 50 μM. Genotoxicity tests have shown that thymol concentration from 20 to 100 μM induced DNA damage in a dose-dependent manner compared to control cells. Figure 7 presents a summary of thymol antineoplastic activity pathways.

## 7. New Forms of Therapeutics Using Thyme Essential Oil and Its Active Ingredients

In order to take advantage of the therapeutic activity of both synthetic and natural substances, an effective way to deliver them to the target cells of the disease should be found. EOs and their ingredients may have irritating properties and may be inactivated in contact with body enzymes. One way to protect the EO ingredients from inactivation after *per os* administration may be the usage of innovative lipid, glycerol, or glycol nanocapsules [78].

### 7.1. Natural Polymers

The research group led by Piombino [79] has shown that it is also possible to encapsulate thymol and its derivatives into microcapsules made of natural polymers. Good biocompatibility with thymol is shown by lignin derivatives, especially lignosulfonate. The synergistic effect of a polyphenolic matrix (lignin derivative) and thymol, as a phenolic active substance, is promising. In addition, lignosulfonate, like thymol, has antioxidant properties. Thymol and its synthetic derivatives were successfully placed in microvesicles with an efficiency up to 50%. A combination of thymol and lignosulfonate was used on the controlled release system and slow-release kinetics of the active compounds were found, which are due to their high lipophilicity. Chen Y. and co-workers [80] examined the antibacterial activity and possibilities of sustained thymol release from porous fibrous membrane made of cellulose acetate. Different thymol membrane loads were used and the results were compared with non-porous membranes. Slower initial release and extended total thymol release time in porous membranes were obtained. The highly developed porous membrane surface and the increase of the entire system’s hydrophobicity enabled more effective release of the substance. Thymol enclosed in porous membrane vesicles showed stronger antibacterial and growth inhibitory activity on *S. aureus* and *E. coli*. The highest activity was obtained with thymol concentration of 15% (*v*/*v*), where the survival rate of these bacteria was 0.07% for *S. aureus* and 0.09% for *E. coli*. The authors point out the possibility of future use of this formulation in wound healing materials.

### 7.2. Semi-Synthetic Polymers

In the research conducted by Pinna [78] on the new formulations of thyme EO, the relationship between the antimicrobial effect of the EO and the structure of carrier polymer nanovehicles was observed. *T. capitatus* EO encapsulated in liposomes was not effective against *Streptococcus mutans* and *C. albicans*, as it did not inhibit the growth of the microorganisms in the tested concentration range. Glyceroliposomes and propylene glycol vesicles containing penetration enhancer (PG-PEVs) with thyme essential oil were active against *S. mutans* (MIC < 0.078 mg/mL), but had no effect on *C. albicans*. No morphological changes were noted after the application of liposomes on *S. mutans*, while glycerosomes caused a characteristic increase in width between the cell wall and the membrane. After contact with PG-PEV vesicles, perforation of the wall and membrane appeared in pathogen cells. Encapsulation of thymol as the active ingredient of thyme EO into microspheres of natural polymers can potentially improve its delivery to the body and compensate poor absorption caused by low water solubility (hydrophobic structure). It also facilitates its oral use by masking taste and reducing irritation. In the study conducted by Rassu and co-workers [81] microspheres based on methylcellulose and phthalate hydroxypropylmethylcellulose (HPMCP) were used. Although encapsulation into methylcellulose microspheres changes thymol pharmacokinetics—shortening its half-life—it causes a significant increase in bioavailability compared to the free thymol form. This form may be beneficial during oral thymol administration to obtain its systemic effect. HPMCP-based microcapsules were poorly absorbed, so they could be used in the treatment of intestinal infections topically. It is important to select suitable polymers, as well as their quantitative ratio. In order to control thymol release, Zamani and co-workers [82] used mixtures of hydroxypropyl methylcellulose with ethylcellulose in various ratios. Microspheres of these polymers in a 5:1 ratio (methylcellulose: ethylcellulose) proved to be the most effective, as they enabled the largest load of vesicles with the active substance and most effectively released it at the destination.

### 7.3. Microfibres

Gámez and co-workers [83] also studied thymol for use in dressings to avoid infections that are a major impediment to the treatment of difficult-to-heal, chronic wounds. Incorporation of controlled-release antibacterial drugs into the dressing is beneficial for wound therapy. During studies on the advanced dressing, mesoporous silicon dioxide loaded with thymol was incorporated into electrospun polycaprolactone microfibers (PCL). Tests conducted with dressing containing 5.6% thymol were carried out using S. aureus, showing that this monoterpene prevented the initial colonization of bacteria and, if it did occur, inhibited their growth and reduced inflammation. The MIC and MBC (minimum bactericidal concentration) values of mesoporous silicon dioxide particles loaded with thymol were significantly lower than those previously found for the free form of thymol. Increased thymol activity has been attributed to slower volatility, increased chemical stability, solubility, and controlled release. Natural, semi-synthetic and synthetic polymers are widely used as carriers of active substances, including in sustained-release models. Therefore, further research and improvement of techniques for obtaining therapeutically effective microvesicles are needed [82,84].

## 8. Conclusions

Current research confirms the broad spectrum of biological and therapeutic effects of thymol and thyme EO. Antibiofilm action includes bacteria such as *E. coli*, *L. monocytogenes*, *P. putida*, *S. aureus* and (MRSA) methicillin-resistant *S. aureus*. Antifungal properties have been demonstrated for *Fusarium* spp., *Aspergillus* spp., *Candida* spp., as well as *C. neoformans* and *C. laurentii*, which were distinguished by resistance to standard antifungal therapy due to their biofilm-forming ability. Activity against parasites such as *L. infantum* has also been observed. Thyme EO and extracts show diverse antiviral activity against such viruses as influenza virus, HSV-1, HSV-2, and HIV-1. In silico studies on the anti-Sars-CoV-2 activity of thymol are also very promising. Unfortunately, the molecular mechanism of action for most of them is not well known. Further research is needed, particularly in vivo, and the therapeutic potential of thyme EO and thymol in infectious diseases should be identified. Thymol also has promising anti-tumor activity through various mechanisms, e.g., associated with the induction of apoptosis and inhibition of proliferation. New forms of medicines with thymol or thyme EO, such as nanocapsules, can be very helpful in therapeutic applications, especially when administered orally, but require further, in-depth research. These studies seem to be promising.
